# Difference Between Intentional and Reactive Movement in Side-Steps: Patterns of Temporal Structure and Force Exertion

**DOI:** 10.3389/fpsyg.2020.02186

**Published:** 2020-09-02

**Authors:** Tsubasa Wakatsuki, Norimasa Yamada

**Affiliations:** ^1^Graduate School of Health and Sport Sciences, Chukyo University, Toyota, Japan; ^2^School of Health and Sport Sciences, Chukyo University, Toyota, Japan

**Keywords:** internally initiated movement, externally triggered movement, whole body, kinetics, onset time, movement time

## Abstract

Intentional and reactive movements are dissimilar in terms of execution time. Previous studies reported that reactive movements are faster than intentional movements (“Bohr’s law” or “Gunslinger effect”), however, these studies focused only on hand-reaching tasks, such as pressing buttons. No studies assessed whole-body movements involving movement of the center of mass (CoM). This movement is characterized by many degrees of freedom because it involves many joints and requires more force than the hand-reaching movement. In this study, we determined the differences in the patterns of temporal structure and force exertion to elucidate the mechanism of “Bohr’s law” in whole-body movement involving movement of the CoM. Ten participants performed a sidestepping task, which requires at least two steps: (1) an intentional movement, in which the movement started with the participants’ own timing; and (2) a reactive movement, in which the movement started the moment a light-emitting diode bulb in front of the participants lit up. We collected data on the ground reaction forces and coordinates of 20 body points. The time of movement onset was calculated and defined based on the ground reaction force, which has the earliest onset compared with velocity and position. The execution time was significantly shorter in the reactive movement condition than in the intentional movement condition (772 vs. 715 ms, *p* = 2.9 × 10^–4^). We confirmed that Bohr’s law was applicable not only in hand-reaching tasks but also in whole-body movement. Moreover, we identified three phases, including the velocity reversal phenomenon associated with the produced mechanism of Bohr’s law, and provided the temporal structure. The difference in the pattern of force exertion accompanying the two styles of motor planning with different accuracies was strongly associated with this motor characteristic. These findings may serve as important basic data to scientifically clarify the mechanism of complex physical tactics implemented in one-on-one dueling in various sports.

## Introduction

One-on-one dueling in sports involves complex physical tactics that rely upon cognitive factors such as decision-making (Cañal-Bruland, 2009; Tsutsui et al., 2019), deception (Brault et al., 2010, 2012), and anticipation (Savelsbergh et al., 2005; Fujii et al., 2014a). The first step to deciphering this complex system is to simplify one-on-one dueling to the greatest extent possible and approach the motor control mechanisms of both participants. The unique study by Welchman et al. (2010) on gunfight scenarios in cowboy films is a representative study of successful simplification of one-on-one dueling. These confrontations are differentiated from those of an attacker and a defender based on who initiates motions. Applying these roles to Welchman et al.’s study, the attacker engages in intentional movement and the defender engages in reactive movement.

Intentional movement involves movement when one decides to move (internally initiated), and reactive movement involves movement in reaction to some external stimuli, such as light and sound (externally triggered). In recent years, the differences in these movements have gained attention in many research fields, including neurophysiology (Kurata and Tanji, 1985; Romo and Schultz, 1987; Mushiake et al., 1991; Maimon and Assad, 2006), neuroscience focusing on brain functions (Deiber et al., 1999; Jenkins et al., 2000; Cunnington et al., 2002; Waszak et al., 2005), electrophysiology focusing on the activity of muscles (Obhi and Haggard, 2004), and medical research for Parkinson’s disease (Jahanshahi et al., 1995; Siegert et al., 2002). These studies suggested that these movements have a different neural basis (Hughes et al., 2011). For example, the pre-supplementary motor area is activated earlier and more significantly in cases of intentional movement than in reactive movement (Cunnington et al., 2002; Soon et al., 2008). The differences in the neural basis also lead to asymmetry in the time taken to execute a movement (movement time: MT). Many studies in experimental psychology that focus on the difference in the MTs of these movements began with the proposal by Nobel laureate, Niels Bohr (1885–1962). He was interested in cowboy films, and he wondered about the portrayal of a hero winning despite a villain moving first in a gunfight. Anecdotal reports suggest that Bohr tested this idea with colleague George Gamow (1904–1968) using toy pistols, with Bohr apparently winning every duel as reactor (= hero) (Cline, 1987). To test Bohr’s proposal, Welchman et al. (2010) devised a unique experiment in which three buttons were pressed in order as fast as possible and measured the MT of a villain (initiator) and a hero (reactor). As a result, they discovered experimentally that the MT of the reactor was approximately 21 ms shorter than that of the initiator. Subsequently, this motor characteristic was named “Bohr’s law” (Pinto et al., 2011) or the “Gunslinger effect” (La Delfa et al., 2013). However, it was concluded that the reactors rarely beat the initiators as in cowboy films because the reactors required approximately 200 ms to react to a visual stimulus.

Although the resolution of Bohr’s proposal was not obtained, the findings from the simplification of a gunfight contributed to simplification of one-on-one dueling in sports with complex structures, especially in ballgames such as basketball, in which the attacker and defender are clearly identified. The first study to relate Bohr’s law to sport was that of Martinez de Quel and Bennet (2014). They adopted the karate punch (“tsuki” in Japanese), which is a specialized motion, and evaluated the MT of intentional and reactive movements. This study confirmed Bohr’s law even in specialized motion. However, its time difference (hereinafter, referred to as reactive advantage) did not exceed the minimum reaction time against visual stimulus, similar to that reported by Welchman et al. (2010). If so, how are defense players able to stop offense players in one-on-one dueling in the context of a ballgame? A previous study examined this issue by categorizing strategies of a defender in basketball into three patterns (Fujii et al., 2014b); nevertheless, we will examine it in terms of execution time for both movements.

To address this question, we focused on the adopted task and on how to define the starting time of a movement. First, all studies relevant to Bohr’s law have adopted a relative motion against the center of mass (hand-reaching task), as in cases of pressing buttons (Welchman et al., 2010; Pinto et al., 2011; La Delfa et al., 2013; Roberts et al., 2017; Weller et al., 2018) and throwing karate punches (Martinez de Quel and Bennet, 2014). These tasks have few degrees of freedom because there are only a few joints involved in the movement. However, in actual sports situations, whole-body movements involving many joints are often performed; therefore, these movements have many degrees of freedom. Many different strategies for quick movement can be applied in case of movements involving many degrees of freedom. Furthermore, whole-body movements involving movement of the center of mass (CoM) require greater force because the whole body has a larger mass than the upper limbs only. Importantly, Oulasvirta et al. (2013) stated that a whole-body movement has a higher degree of difficulty in motor control than hand-reaching movement, and examined the information capacity of whole-body movement. Previous studies did not observe the forces that generate these movements. Examining the temporal changes in force and velocity, and the relationship of the CoM and external force in whole-body movement with the movement of the CoM will allow us to elucidate the mechanism of Bohr’s law. Second, previous studies defined the starting time of movement (hereinafter, referred to as onset time) based on “ON” or “OFF” of the electric signals by pressing a button (Welchman et al., 2010; Pinto et al., 2011; La Delfa et al., 2013; Roberts et al., 2017; Weller et al., 2018), or the velocity of a specific body part (Martinez de Quel and Bennet, 2014) because of the simplicity of the concept. However, using these methods, the displacement of the body coordinates had already started because there is a phase difference between velocity, acceleration, and position. The movement onset velocity or the movement to press each button is the conclusive outcome assessed by the exerted force related to the movement, and a time delay occurs between when the force is exerted and the defined onset time. Therefore, we hypothesized that if these two aspects were improved, the results would be different from those of the previous studies, meaning the reactive advantage would exceed 200 ms.

In this study, we assessed a sidestepping task that requires at least two steps involving the movement of the CoM and calculated the MT using onset time obtained based on the ground reaction force (GRF), to verify whether Bohr’s law is applied and whether its reactive advantage exceeds the minimum reaction time against visual stimuli. We also measured the difference in patterns of temporal structure and force exertion between intentional movement and reactive movement and evaluated the produced mechanism of Bohr’s law that is applied during force generation to displace the body coordinates. The adopted task is similar to a task adopted in Welchman et al.’s (2010) study in that it is not a single-moment movement (e.g., vertical jump). Finally, we considered it to be more specialized and characterized by a more difficult level of motor control than a karate punch because the subject controls the right and left legs separately, and there is an aerial phase in which both feet are off the ground.

## Materials and Methods

### Participants

Ten healthy male subjects participated in this study [age = 22 ± 1.1 years, height = 176.5 ± 8.3 cm, weight = 73.9 ± 10.0 kg (mean ± SD)]. All participants were experienced in basketball, handball, or football. All often performed sidestepping and therefore were accustomed to the movement [experience = 10.6 ± 2.1 years (mean ± SD)]. All participants had normal or corrected-to-normal vision. Table 1 presents participants’ physical characteristics. The experimental procedures were conducted in accordance with the Declaration of Helsinki and were approved by the ethics committee of Chukyo University (approval number: 2017–046). Before conducting the experiment, the purpose of the study and experimental protocols were explained to the participants. Written informed consent was obtained from all participants.

**TABLE 1:** Physical characteristic of all participants.

**Participant ID**	**Age (years)**	**Height (cm)**	**Weight (kg)**	**Specialized sports**	**Years of experience**
1	24	170.5	54.1	Football	10
2	24	178.2	83.2	Basketball	16
3	22	162.3	58.7	Basketball	8
4	21	174.5	87.7	Handball	11
5	21	178.6	75.9	Handball	11
6	22	177.6	72.3	Handball	11
7	21	168.0	79.1	Handball	11
8	21	177.5	76.3	Handball	8
9	22	183.2	71.0	Basketball	10
10	22	194.2	80.5	Basketball	10
Total (mean ± SD)	22 ± 1.1	176.5 ± 8.3	73.9 ± 10.0		10.6 ± 2.1

### Protocol and Apparatus

The participants performed a sidestepping task, which required at least two steps. The participants stood on two force plates that were embedded in the ground with each foot on a different plate in a comfortable position, balancing their weight evenly on both feet. They were instructed to take steps laterally toward the right from the static state and reach the target line marked at a distance equal to their own body height from the initial midline, as fast as possible.

**FIGURE 1:**
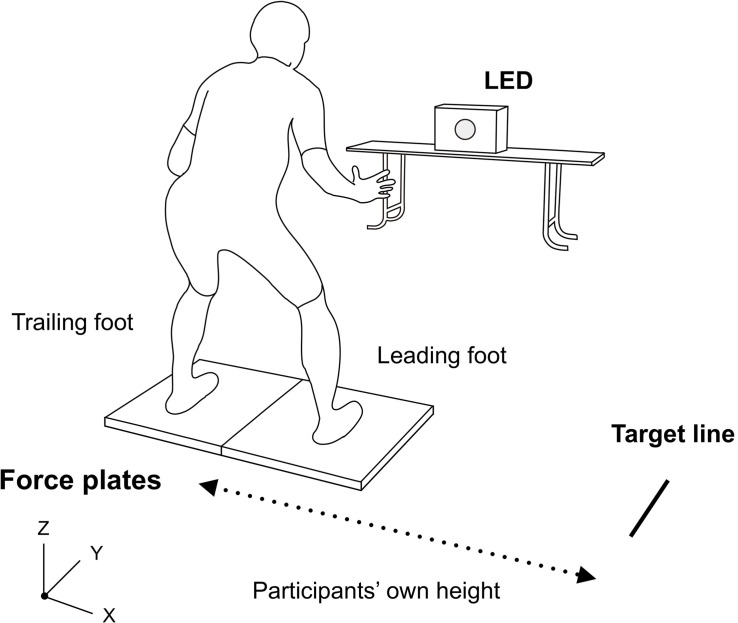
Experimental setup. Participants prepare by standing with one foot each on the two force plates. Thereafter, they take steps laterally toward (“x” component) the right and reach the marked target line at a distance equal to their own body height. The light-emitting diode bulb placed in front lit up only under the reactive movement condition (RMC).

Two conditions were studied in this experiment: an intentional movement condition (IMC) and a reactive movement condition (RMC). In the IMC, each participant started moving on the participant’s own timing. In the RMC, each participant started moving when a light-emitting diode (LED) bulb placed in front lit up (Figure 1). Under both conditions, there was no regulation as to motion or posture (e.g., number of steps, joints angle). After five practice trials, the participants completed 10 trials for each condition (total 20 trials). To avoid getting accustomed to the continuation of the same conditions, the two conditions were conducted alternatively for one trial each (IMC1 → RMC1 → IMC2 → RMC2…IMC10 → RMC10). The participants were informed of the subsequent conditions before the trial. To eliminate the effect of fatigue, participants were given more than 2 min to rest between trials.

Three-dimensional coordinates of the landmark points were acquired using a 3D optical motion capture system with 10 cameras at 250 Hz (Vicon MX; Vicon Motion Systems, United Kingdom). Twenty reflective markers were placed on each participant’s body (i.e., their head and upper margin of sternum, right and left side of their ears, shoulders, elbows, wrists, hips, knees, ankles, heels, and toes). All raw coordinate data points were smoothed using a fourth-order Butterworth low-pass filter, with a cut-off frequency of 25 Hz. In addition, to measure the GRF on both legs, two force plates at 1000 Hz were used (9287C; Kistler, Switzerland). The coordinates data collected at 250 Hz were linearly interpolated to 1000 Hz for the analyses.

### Data Analysis

#### Performance Variables in Sidestepping

The performance variables in sidestepping were defined as follows: (1) movement time (MT), the time from onset time to reach time. The onset time was calculated based on the following two timings, whichever comes first, i.e., the time to the vertical force (F_*z*_) for the trailing foot increasing to > 10% of the static state, and the time to the F_*z*_ for the leading foot decreasing to < 10% of the static state. The reach time was the time recorded when the lateral torso displacement was 60% of their own body height, according to Fujii et al. (2013); (2) lateral peak velocity, the peak value of the torso velocity in the “x” component (V_*x*_); (3) time to peak velocity, the time from the onset time to the instant at which V_*x*_ reaches its peak; and (4) lateral peak force, which is the peak lateral GRF (F_*x*_) of the trailing foot in the first step. The CoM of the torso was calculated based on an estimation of the body segment parameters (Ae et al., 1992). All numerical calculations, including the analyses, were performed using Mathematica 10 (Wolfram Research, IL, United States).

#### Statistical Analysis

Trials were eliminated from the analysis as error trials when the MT exceeded the average ± 2 SD for each participant or if there was difficulty in detecting the onset time because of sway of the GRF. To standardize the number of trials for analysis for all participants, the number of trials for the participant with the smallest number of remaining trials (i.e., the most error trials) was adopted. The four performance variables in these trials were compared between the IMC and RMC using a paired *t*-test, and were also compared within participants using a paired *t*-test. For statistical calculations, *p* < 0.05 was considered significant. The statistical analyses were performed using Mathematica 10 (Wolfram Research, IL, United States).

## Results

### Error Trials

Of 100 trials (10 trials × 10 participants) in each condition, there were 10 error trials in IMC and 6 error trials in RMC. The number of trials for the participant with the smallest number of remained trials was seven. Based on this result, we adopted the initial seven trials of each participant as the trials for analysis and eliminated the other trials. Therefore, the final number of remained trials for analysis was 70 trials (7 trials × 10 participants) for each condition.

### Sidestepping Motion

The onset time of movement is defined in two ways, i.e., the weighting of the trailing foot (GRF of left side) or the unweighting of the leading foot (GRF of right side), whichever comes first (see section “Performance Variables in Sidestepping”). In terms of which came first in the actual results, for the IMC, 80% of all trials (56/70 trials) were performed by the unweighted leading foot first (the weighted trials of the trailing foot first were the remaining 20%). By contrast, there was a 50–50 split in the RMC (one trial only had the same time). Confirming the individual results, there was a mix of the participants using two different initiation patterns depending on the conditions, the participants consistently using the same initiation patterns, and the participants using random patterns. However, in the IMC, it was common for all participants to have more trials in which the unweighted state of the leading foot was first.

**FIGURE 2:**
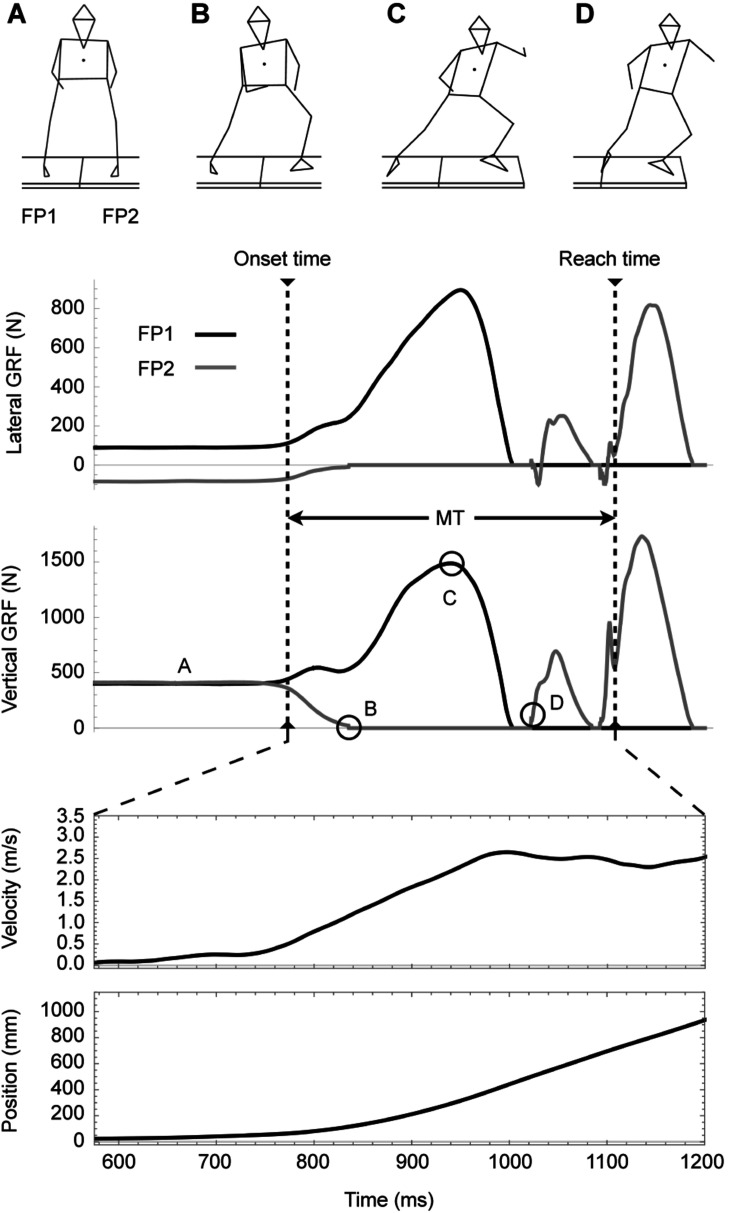
Typical example of sidestepping motion and characteristic phases (viewpoint: back). From the top, lateral ground reaction force (GRF; F_*x*_), vertical GRF (F_*z*_), lateral velocity, and lateral position. Phase **(A)** is the preparation phase, phase **(B)** is the takeoff of the leading foot, phase **(C)** involves reaching the peak force for the trailing foot, and phase **(D)** involves landing of the leading foot. These data represent one of the IMC results.

Figure 2 displays a typical example of the GRFs (lateral and vertical), lateral velocity, lateral position, and characteristic four phases of sidestepping motion. This figure contains an example of the IMC result. FP1 (black line) is the force plate with a left foot (trailing foot) on before moving, and FP2 (gray line) is the force plate with a right foot (leading foot) on. This figure shows that the velocity and position have not changed yet, although the forces have already risen.

Phase A is preparation, phase B is takeoff of leading foot, phase C is reaching peak force of trailing foot, and phase D is landing leading foot. Between C and D, there is a both-foot takeoff phase where the two force plates indicate zero, meaning the transition phase between the first and second steps, as well as the maximum velocity reaching point of the first step (around 1000 ms in this figure). The maximum value of the FP2 is the moment when the trailing foot lands for the second step. Furthermore, this figure also shows the onset time and reach time required to calculate the MT; the maximum value of FP2 appeared after reach time.

### Performance Variables in Sidestepping

**FIGURE 3:**
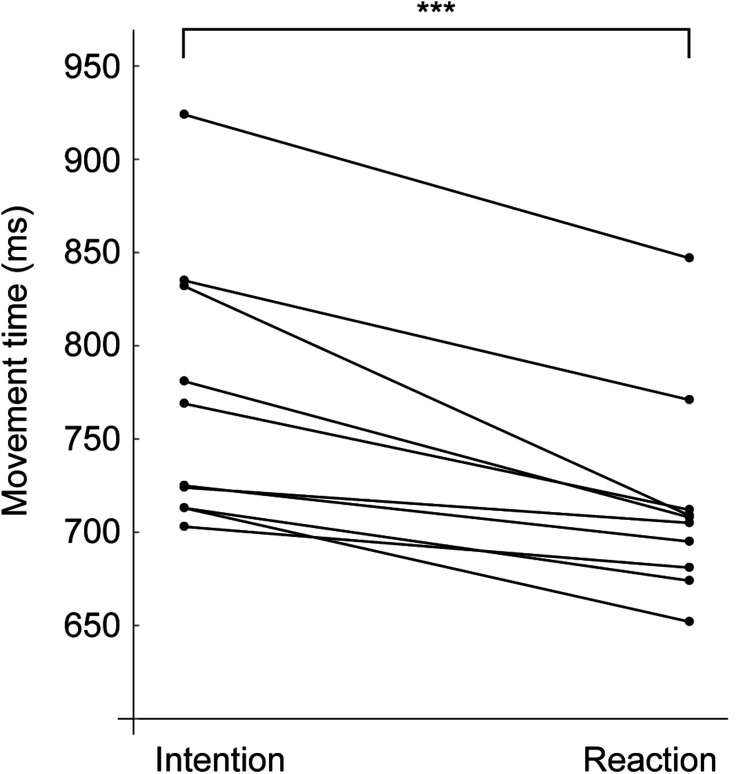
Movement time for each participant. Points connected by lines indicate the average from a single individual. The reaction is shorter than the intention in all participants, and there is a significant difference between two conditions (*p* = 2.9 × 10^–4^). ***p < 0.001.

**FIGURE 4:**
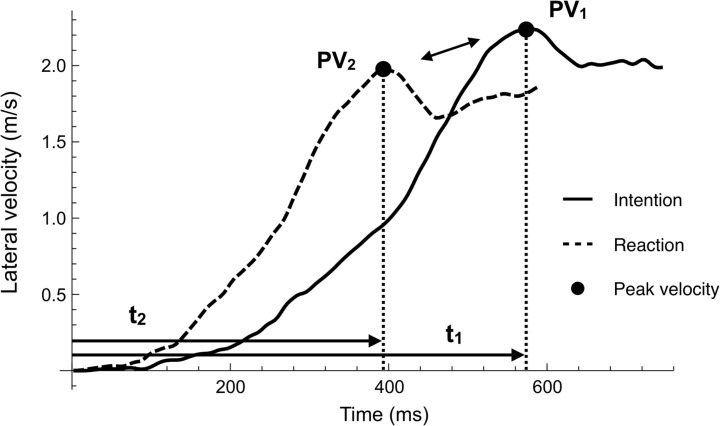
A typical example of lateral velocity (“x” component) in two conditions. The peak velocity (black dots) indicates the moments for the takeoff of both legs in the first step. The intentional movement (solid line) slowly reaches the peak velocity and a gentle curve is obtained, whereas the reactive movement (dashed line) is linear. The time difference (*t*_1_–*t*_2_) generated until the peak velocity is reached is largely related to the final MT difference.

Table 2 presents the performance variables. (1) Movement time (MT) for the RMC was significantly shorter than that for the IMC (772 ± 72 vs. 715 ± 56 ms, *p* = 2.9 × 10^–4^, *t*_9_ = 5.71, *d* = 0.88). The mean reactive advantage was 57 ms. On assessment for within-participant differences in conditions, a significant difference was found in 8 of 10 participants (all *ps* < 0.05 and *ds* > 0.8), with no significant differences in the remaining two participants (all *ps* > 0.05); however, there were no participants in whom MT in RMC exceeded that in IMC (Figure 3). The MT in RMC was significantly shorter than that in IMC, not only in relative distance (to 60% of height) but also in absolute distance (to 50 cm) (537 ± 48 vs. 481 ± 37 ms, *p* = 1.4 × 10^–4^, *t*_9_ = 6.28, *d* = 1.31). (2) For lateral peak velocity, the IMC was significantly greater than the RMC (2.55 ± 0.25 vs. 2.45 ± 0.29 m/s, *p* = 8.4 × 10^–5^, *t*_9_ = 6.74, *d* = 0.37). On assessment for within-participant differences in conditions, a significant difference was found in only 5 of 10 participants (all *ps* < 0.05 and *ds* > 0.8), and the remaining 5 participants showed no significant differences (all *ps* > 0.05), however, the lateral peak velocity in IMC exceeded that in RMC in all participants. (3) For time to lateral peak velocity, the RMC was significantly shorter than the IMC (511 ± 65 vs. 427 ± 43 ms, *p* = 1.4 × 10^–5^, *t*_9_ = 8.44, *d* = 1.5). On assessment for within-participant differences in conditions, a significant difference was found in all participants (all *ps* < 0.05 and *ds* > 0.8). Interestingly, the difference between conditions in time to lateral peak velocity exceeded the condition difference in MT. Specifically, the time difference in MT was 57 ms (772–715 ms) and the time difference in time to peak velocity was 84 ms (511–427 ms). This trend was confirmed for all participants. Figure 4 shows a typical example of the relationship between the peak value of lateral velocity (V_*x*_) and the time to reach it. This figure shows that an intentional movement (solid line) slowly reaches a peak while drawing a gentle curve, whereas a reactive movement (dashed line) quickly reaches a peak in a linear manner. Looking at the figure in a time series demonstrated that the RMC, which led the position from the onset, first reached the peak velocity, and thereafter, the IMC, slowly increased the velocity, and subsequently reversed the RMC. Finally, (4) lateral peak force, there was no significant difference between the two conditions (747 ± 126 vs. 731 ± 129 N, *p* = 0.14, *t*_9_ = 1.61, *d* = 0.13). Table 3 presents the performance variables of each participant.

**TABLE 2:** Performance variables in sidestepping.

**Variable**	**Intention (*n* = 10)**		**Reaction (*n* = 10)**		**Significance**
	**M**	**SD**	**M**	**SD**	
MT (ms)	772	72	715	56	***
Lateral peak velocity (m/s)	2.55	0.25	2.45	0.29	***
Time to lateral peak velocity (ms)	511	65	427	43	***
Lateral peak force (N)	747	126	731	129	n.s.

Statistical significances of the difference between the two conditions: ***p < 0.001.

**TABLE 3:** Performance variables of each participant (mean ± SD).

**Participant ID**	**Condition**	**Performance variables**							
		**MT (ms)**		**Lateral peak velocity (m/s)**		**Time to lateral peak velocity (ms)**		**Lateral peak force (N)**	
1	Intention	835 ± 46	*	2.28 ± 0.10	***	538 ± 39	***	505 ± 38	n.s.
	Reaction	771 ± 10		2.12 ± 0.09		422 ± 15		471 ± 19	
2	Intention	713 ± 23	*	2.69 ± 0.06	n.s.	444 ± 25	**	911 ± 28	n.s.
	Reaction	674 ± 14		2.59 ± 0.10		380 ± 17		902 ± 27	
3	Intention	703 ± 28	*	2.55 ± 0.04	*	497 ± 31	**	610 ± 22	n.s.
	Reaction	681 ± 14		2.39 ± 0.12		448 ± 13		588 ± 30	
4	Intention	725 ± 23	n.s.	2.57 ± 0.08	n.s.	470 ± 26	*	782 ± 75	n.s.
	Reaction	695 ± 38		2.51 ± 0.10		423 ± 29		800 ± 51	
5	Intention	781 ± 24	***	2.93 ± 0.04	n.s.	572 ± 26	***	712 ± 18	n.s.
	Reaction	708 ± 11		2.91 ± 0.04		475 ± 18		714 ± 28	
6	Intention	713 ± 43	**	2.89 ± 0.04	*	502 ± 48	***	708 ± 25	n.s.
	Reaction	652 ± 37		2.81 ± 0.08		421 ± 30		714 ± 48	
7	Intention	769 ± 45	**	2.20 ± 0.13	*	468 ± 58	**	779 ± 46	*
	Reaction	712 ± 10		2.05 ± 0.08		386 ± 19		747 ± 31	
8	Intention	724 ± 25	n.s.	2.37 ± 0.13	n.s.	416 ± 18	**	860 ± 53	n.s.
	Reaction	705 ± 9		2.25 ± 0.10		365 ± 12		878 ± 47	
9	Intention	832 ± 80	*	2.68 ± 0.08	n.s.	583 ± 82	*	891 ± 59	*
	Reaction	709 ± 41		2.61 ± 0.07		447 ± 48		801 ± 42	
10	Intention	924 ± 43	**	2.33 ± 0.07	*	616 ± 44	**	713 ± 40	n.s.
	Reaction	847 ± 22		2.25 ± 0.03		503 ± 32		693 ± 46	

Statistical significances of the difference between the two conditions: *p < 0.05; **p < 0.01; ***p < 0.001.

## Discussion

### “Bohr’s Law” in Side-Steps

The main variable to examine the presence or absence of Bohr’s law is MT, and this variable was significantly shorter in the RMC than in the IMC. Therefore, we confirmed again that Bohr’s law applies not only in hand-reaching tasks, such as the pressing of a button or the punching motion in karate, but also in whole-body movements with moving the center of mass, such as in side-steps. The supplemental result was that the MT of RMC was significantly shorter than MT of IMC not only in terms of relative distance but also in terms of absolute distance, suggesting that this motor characteristic is independent of height. We defined the onset time using the force that was exerted on the ground (GRF) instead of the displacement of the body coordinates. This new method means that the measurement of MT started before displacement of the body coordinate started. The confirmation of “Bohr’s law” in this method provides strong support for the hypothesis that these two movements have different neural basis (Hughes et al., 2011). However, as reported by Welchman et al. (2010) in our study, the reactive advantage was not great enough to cover the potential disadvantage of the reactors (mean 57 vs. approximately 200 ms). Therefore, we suggest that there is another explanation for success of the defense in ballgames such as basketball where the offensive player makes an intentional movement and the defensive player makes a reactive movement. Much of this mechanism may be explained by the anticipation capacity that is supposedly inherent in humans regardless of their specialized experience in sporting events (Fujii et al., 2014a). The reactive advantage (time difference by Bohr’s law) generated in whole-body movement may have a role to play in assisting it.

### Difference in the Patterns of Force Exertion

Because the movements adopted in this experiment had MT of less than 1 s, and these were simple tasks without a choice process, we had predicted that the difference in lateral peak velocity would be a major factor. In other words, we had hypothesized that the greater the lateral peak velocity, the shorter the MT would be. However, although the MT in RMC was shorter than that in IMC, the lateral peak velocity in RMC was not greater than that in IMC.

The peak velocity reaching point corresponds to the moment of takeoff of both feet, and this moment is between phases C and D in Figure 2, in which both feet indicate zero. Because the peak velocity, that is, the velocity at takeoff, is determined by the impulse (product of force and time), the fact that there is no significant difference in the lateral peak force (although there is a significant difference in the lateral peak velocity) suggests that there is a difference in time of applying force. Therefore, we believe that the difference in the patterns of force exertion until the reaching lateral peak velocity is a factor that causes asymmetry in MT (i.e., Bohr’s law). In support of this proposal, the time to lateral peak velocity in RMC was significantly shorter than that in IMC for all participants, and this result is consistent with that of the study by Martinez de Quel and Bennet (2014). This variable appears to have a great effect on the production of reactive advantage in Bohr’s law.

To clarify this viewpoint, Figure 4 displays a typical example of lateral velocity. This figure shows that the intentional movement slowly reaches the peak while drawing a gentle curve, whereas the reactive movement is linear in a significantly shortened time. Interestingly, the difference in time to lateral peak velocity always exceeded the difference in final MT. This finding suggests that the reactive advantage is not produced after the peak velocity is reached, meaning that the velocity of the intentional movement reverses the velocity of the reactive movement. The finding supports a report that examined the boundary condition of this motor characteristic, stating that the overall reactive advantage decreased as more steps were added (Pinto et al., 2011). For example, the difference in the MT in a two-step task was less than that in a one-step task. Similarly, Welchman et al. (2010) adopted an experiment using three buttons and reported that the advantage of the reaction produced in the first step (button 1 to button 2) was reduced in the second step (button 2 to button 3), and was not produced in the third step (button 3 to button 1). This may be explained by the execution of the second step becoming more similar across conditions.

### Temporal Structure of “Bohr’s Law”

Although the lateral peak velocity in RMC is not greater than that in IMC, the final MT is less in RMC than that in IMC. It is likely caused by the shortening of the time to lateral peak velocity. The temporal relationship between the MT and time to lateral peak velocity suggests that the lateral velocity is reversed during movement. Therefore, the initiator who exerts greater velocity chases the reactor who quickly reaches the peak velocity due to the explosive start, however, because the difference in time to peak velocity is influenced, the reactor finally completes the task in a shorter time.

**FIGURE 5:**
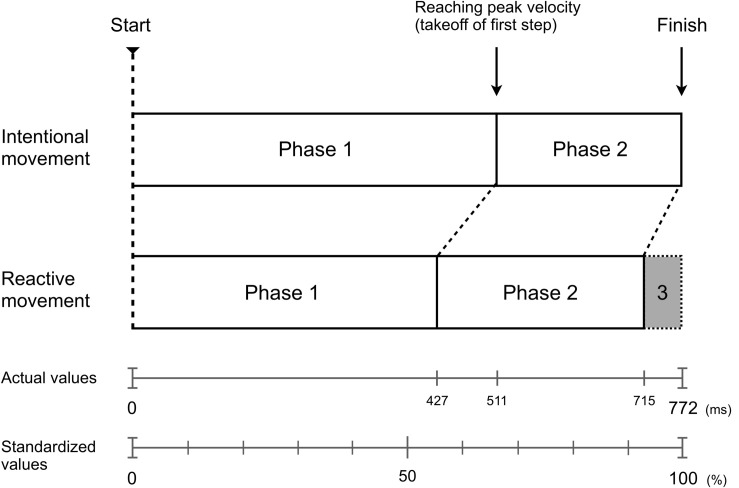
Conceptual scheme of the temporal structure according to the three phases including the velocity reversal phenomenon. The shaded gray area shows the conclusive generated time difference in the MT (reactive advantage). Phase 1: the reactive movement leads the intentional movement until the peak velocity is reached, generating greater differences in the MT (reaction advantage phase). Phase 2: the intentional movement, which generated a greater peak velocity than reactive movement, reduced the difference in the moving distance from the reactive movement (intention advantage phase). Phase 3: the reactive movement holds the difference in the MT generated until the peak velocity is reached, leading to a difference in the final MT (still intention moving phase). The scale below shows the actual values, and the standardized value when the MT of IMC is 100 (all values are average).

Accordingly, we divided the mechanism of producing Bohr’s law into three temporal characteristic phases. In the first phase, the reactor generates an overwhelming difference in the MT (reaction advantage phase). In the second phase, the initiator reduces the difference in the moving distance due to the velocity reversal phenomenon (intention advantage phase). In the final phase, the reactor holds the generated first phase difference in the MT and produces a difference in the final MT (still intention moving phase). Figure 5 shows a conceptual scheme of the temporal structure based on the three phases. This structure characterizes the temporal mechanism of Bohr’s law.

### Motor Control Styles of Both Movements

The three phases were characterized by structural mechanisms in which Bohr’s law was applied due to the difference in the force exertion pattern and the velocity reversal phenomenon. Here, we discuss this phenomenon focusing on the accuracy of motor planning and the motor control styles.

Generally, the motor control style is divided into the feedforward and feedback control styles. Many studies on these control patterns have been conducted in the past. Among them, the study by Woodworth (1899) is the oldest, however, it is an important idea for considering the two styles in terms of the patterns of force exertion. Woodworth (1899) compared the trajectory of high-frequency motion and low-frequency motion using the drawing line task with a pen and distinguished between the two control styles and named each them “initial impulse” and “current control.” This has led to the current research on the feedforward and feedback control styles.

Now it is assumed that the feedforward control, i.e., initial impulse, is used in the initiation of sidestepping, adopted in this study. The major difference between intentional and reactive movement is in the first phase (reaction advantage phase) until the peak velocity is reached; we confirmed that the peak velocity was achieved faster in reactive movement than in intentional movement. An intention can be initiated at the participant’s own timing; thus, it is possible to set “elaborate” motor planning from the start to finish. Because of the trade-off association between speed and accuracy (Fitts, 1954), it is necessary to reduce the velocity at the start to allow for an elaborate motor planning. By contrast, elaborate motor planning cannot be achieved for reactive movements because the trigger is external. Therefore, it is likely that reactive movement involves “coarse” motor planning, and the highest priority is to concentrate on reaching out and reaching the peak velocity as fast as possible. In the next phase (intention advantage phase), after the reactive movement achieves peak velocity, velocity reversal occurs. In this phase, the reactive movement adjusts the movement by decreasing the velocity. Therefore, it is considered that the motor control styles of intentional movement (internally initiated movement) and reactive movement (externally triggered movement) is a further subdivision of the initial impulse proposed by Woodworth (1899), although the accuracy of arrival at the target line was not examined in this study.

For reference, the initiation patterns by the participants (discussed in section “Sidestepping Motion”) may supplement the explanation of these motor control styles. We believe that unweighting the leading foot first means prioritizing preparation for the move over force generation, and weighting the trailing foot first means prioritizing the force generation required to get off to an explosive start. In this study, there were three participants who showed a tendency to use two different initiation patterns depending on the conditions, and they showed a tendency to use the former for the IMC and the latter for the RMC (also importantly they showed no reverse pattern). This may possibly support the earlier result that it is possible that the intentional and reactive movements can further divide the “initial impulse” into two styles.

### Research Limitations

This study has three limitations. The first limitation is the accuracy of the onset time. Calculating the onset time based on the GRF was very difficult because the GRF showed minor fluctuations in a stationary state. The calculation method used in this study was derived from several preliminary experiments and many motion patterns, however, we believe that further validation is necessary. The second limitation is associated with the adopted task. Considering the previous limitation, the sidestepping task used in this study was simplified for experimentation. In actual sport situations, it is rare to suddenly start moving from a stationary state, and many movements involve preparational motion such as the split step in tennis (Uzu et al., 2009). Previous studies have reported that the unweighted state during preparatory motion of the side-step shortens the reaching time (Fujii et al., 2013) and increases the success rate of the defenders (Fujii et al., 2015). However, because calculating the onset time based on the GRF was an original idea in this study, we could not include the preparatory motions for the sidestepping task. The third limitation is the kind of stimuli (signal). We used a LED bulb, which is a digital stimulus in this study, however, the actual human movements are analog stimuli. Therefore, we needed to conduct experiments in an environment where the two subjects faced each other, and we needed to examine the relationship between the two subjects based on their GRFs. However, the GRFs of two subjects could not be measured simultaneously because of the limited number of force plates. In this case, it may also be necessary to increase the degrees of freedom in the moving direction, i.e., to adopt a choice reaction task. Despite these limitations, our findings may provide initial basic data in the process of scientifically clarifying the mechanism of complex physical tactics implemented during one-on-one dueling in various sports.

## Data Availability Statement

All datasets generated for this study are included in the article/supplementary material.

## Ethics Statement

The studies involving human participants were reviewed and approved by the Ethics Committee of Chukyo University. The patients/participants provided their written informed consent to participate in this study.

## Author Contributions

TW and NY conceived and designed the study. TW conducted the experiment, analyzed the data, and wrote the manuscript. NY contributed to the improvement of the manuscript. Both authors contributed to the article and approved the submitted version.

## Conflict of Interest

The authors declare that the research was conducted in the absence of any commercial or financial relationships that could be construed as a potential conflict of interest.
